# Proceedings of the Pathological Society of Philadelphia

**Published:** 1859-11

**Authors:** 


ART. V.—*Proceedings of the Pathological Society of Philadelphia.*

WEDNESDAY EVENING, June 8th, 1859.

DR. HEWSON in the Chair.

DR. DARRACH exhibited a specimen of extensive post-mortem digestion of the stomach, which occurred in a case of tubercular meningitis.

*Tubercle of the Meninges of the Brain and of the Kidney.*—DR. HUTCHINSON said: The specimens which I present were removed from the body of a German, aged twenty-six, who died yesterday afternoon.

He was admitted into the Pennsylvania Hospital, on the 19th of March, for phthisis, which had not then advanced to the stage of softening. Shortly after his admission he complained of difficulty in passing his water. Upon examining the urine it was found to be albuminous, and to contain pus and blood-cells in great abundance. He also complained of pain upon pressure in the region of the kidneys.

He suffered much from severe headache, and from the first evinced a decided tendency to sleep. There was also pharyngitis, but I could see no ulceration.

The history of the case is rather imperfect. Two years ago he had been caught in a severe shower and thoroughly drenched. This circumstance had given rise to a severe cough, which had afterwards assumed a chronic form. He had had night-sweats, and had been losing flesh for some time previously.

His condition did not improve under the ordinary treatment for phthisis. The urine deposited pus and blood, even more copiously than at the time of his entrance into our wards. The pulse was very frequent. No diarrhœa occurred. Ten days ago there was a marked increase of the pain in his head, and delirium set in. During the delirium he was exceedingly restless, and uttered almost constantly short, quick cries of pain. Five days ago he became semi-comatose, and on Sunday complete coma supervened.

Death took place on Tuesday.

The autopsy was made fifteen hours after death. The head was first examined. There was found a slight congestion of the membranes of the brain, and an effusion of four ounces of serum in the ventricles. Tubercles of the size of a pin's head were deposited in the pia mater, especially on each side of the pons Varolii and in the fissure of Silvius. Upon opening the lateral ventricles the septum appeared to be incomplete. Miliary tubercles were scattered through the lungs in great abundance. A small cavity was detected at the top of the right lung.

The left kidney was somewhat larger than normal, and presented a "bosselated" appearance; its whole structure was destroyed and converted into a series of tuberculous abscesses—some of them sufficiently large to contain a walnut. These abscesses showed no tendency to communicate with the abdominal cavity. The right kidney was rather enlarged, and contained a few minute tubercles in its cortical substance. The left ureter was considerably dilated. The bladder was thickened, and had its mucous membrane slightly roughened. I could discover no tubercles either in the peritoneum or in the mesenteric glands. The right testicle had undergone tubercular degeneration.

In two other cases which have happened recently in the hospital, the left has been the kidney affected; and, in referring to M. Rayer's work, I find that out of sixteen cases, the disease has attacked both kidneys six, the left seven, and the right only three times.

In none of these cases did tubercular meningitis exist as a complication.

*Fatty Kidneys in Typhus Fever.*—DR. HUTCHINSON continued: I also present two other kidneys removed from a patient, who during life presented the symptoms of typhus fever, and after death some of the appearances of that disease. One kidney is found to be uncommonly enlarged, double the ordinary size; the other to have undergone complete degeneration. Under the microscope nothing but oil could be discovered. The urine was albuminous, and contained free oil.

The patient was under treatment only a few days. Nothing satisfactory could be learned in regard to her former history, but she seems to have been well two weeks previous to her admission here.

Considerable discussion followed the exhibition of both these specimens.

DR. PACKARD had noticed tubercle corpuscles and some fibrous tissue and spindle-shaped cells in the tubercular kidney. The fibrous tissue and the cells were in the walls of the abscesses.

DR. WOODWARD subsequently reported more particularly on the structure of the walls, and stated that they were mainly composed of unsoftened tubercular matter. The spindle-shaped cells he believed to belong to the lining of the tubuli uriniferi.

*Partial Fractures of the Ulna.*—Presented by DR. LENOX HODGE.

Frederick Trump, aged fifteen, while at work in a machine-shop, had his left arm caught by the band, and was twice carried around the shaft. His arm was torn completely off, about three inches below the shoulder-joint; the skin and tissues around the shoulder were much lacerated.

Amputation was performed by Dr. Norris through the joint; and the patient recovered without a bad symptom.

Upon examining the arm we found that there were, besides the fracture of the humerus, a fracture of the radius, and two partial fractures of the ulna.

The ulna, with its partial fractures, I exhibit to you to-night. The upper is situated at the juncture of the upper and middle third; the lower is in the middle of the lower third of the bone. They both involve the periosteum on the anterior surface, pass obliquely and for a short distance longitudinally upward; the fibres that remain unbroken are bent, so that the whole bone is curved forward. In this specimen there is no *angular* deformity, and when covered by muscles, fasciæ, and skin, would furnish, not the symptoms of partial fracture, as usually given, but of bent bones. "Pain and loss of power in the limb, its preternatural curve, which could be increased or diminished optionally, and the disposition in the bones when straightened to return to their flexed position, unattended by crepitus, or that circumscribed projection which a displaced fragment of bone would produce."* So that in a case like this there would be nothing left to guide us, in forming a diagnosis, but the age of the patient. Indeed, the accident might occur, if the blow or fall were sudden and severe, even in children under two years, and then the partial fracture could not be distinguished from the bent bone. It may be that this has really happened in some instances where the injury was regarded as a bent bone. And since some fibres of the bone must have given way if the curve is reproduced when the force that straightened it is removed, it would perhaps be more accurate and simple to classify, as Dr. Hamilton does,† bent bones as a mere division of incomplete fractures.

Within a few weeks several cases of incomplete fractures have been brought to the Pennsylvania Hospital. One was that of a clavicle of a boy, eight years of age; the curve was forward and downward; was unattended by crepitus or angular deformity. The boy had fallen down several steps, and had at the same time fractured his humerus. The deformity of the clavicle could be but partially removed. Another was that of the forearm of a boy of fifteen years of age, who had fallen down five weeks after a fracture, which he describes as "having gone clean through." The arm was perfectly straight, he says, until he fell. When brought to the hospital, both bones were *curved* very much backward. The middle of

---

* Remarks on certain Injuries of the Bones in Children. By John Rhea Barton, M.D. American Med. Recorder, Jan., 1821, p. 11.

† Report on Deformities after Fractures. By Frank Hastings Hamilton. Transactions American Med. Association, 1855, p. 421.

this curve was about an inch and a half above the wrist. The bones could be straightened, but a part of the deformity had a tendency to return. The present incomplete fracture occupies about the same position as the previous complete fracture. There was but little callus present; so little, indeed, that a previous fracture would not probably have been suspected. This case is mentioned as being analogous to, though perhaps not strictly identical with, an incomplete fracture of *bone*.

This morning, a boy, sixteen years of age was brought to the hospital, with many severe injuries, received by being carried twice around a shaft in a steam marble works. His left forearm is curved forward, on the radial side, gradually throughout its whole length, and bent forward, on the ulnar side, angularly, at about two inches above the wrist. There is no crepitus perceptible, neither in the radius nor ulna. The ulna, indeed, presents the signs of a partial fracture, while the radius exhibits those usually attributed to a bent bone. But owing to the age, and the specimen exhibited to you, I am led to consider them both as partial fractures.

To these I would add another injury, caused by a cart-wheel passing over the forearm, in which the radius was broken, and the ulna presented all the signs of an incomplete fracture. The ulna was bent strongly toward the radial side, but could be straightened. There was a marked tendency toward reproduction of the deformity when the counteracting force was removed. No crepitus could be elicited; there was no increase of mobility except to and from the radial side. There was an angular projection in the upper third of the bone. The man is twenty-six years of age, and apparently healthy and well developed. I have not found a similar case recorded as occurring in an adult; and even Dr. Barton says:* "That a partial fracture cannot occur in a healthy adult, is very possible." Yet there seems to be no good reason why it *may* not occur. The rapidity and force with which the blow is given, the extent of surface to which it is applied, might, it would seem, be so varied as to sever all the fibres or only a portion of almost any given substance. And there are instances recorded† even of old persons (where the earthy constituents preponderate still more) having had partial fractures of the neck of the femur. In the above-mentioned case, notwithstanding all our efforts, there is still now (at the end of five weeks) a perceptible bend toward the radius.

---

* Amer. Med. Recorder, Jan., 1821, p. 14.

† Guy's Hospital Reports, second series, vol. ii. p. 347. American Jour. of Med. Science, April, 1857, p. 306. Fractures of Neck of Thigh-Bone, by R. D. Mussey, M.D. Or, as quoted by Dr. Packard in the American edition of Malgaigne's Treatise on Fractures, Philadelphia, 1859, p. 552. In the case recorded by Dr. Mussey, the patient was only forty-two years of age.

The exhibition of this specimen led to some remarks by Drs. Hewson, Packard, and Hall, on the diagnosis between partial fracture and bent bone.

*Severance of Cervix Uteri during Labor.*—DR. KELLER said: It will be remembered that at a former meeting of the Society, (see page 17 of its Proceedings,) I presented a specimen of a torn-off neck of the uterus. I wish, to-night, to add some additional particulars of the case, and especially to record the fact that the patient has since been delivered of a healthy child, without difficulty.

Mrs. P., twenty-eight years of age, from Bohemia, after having suffered for almost two months from violent spasms of the womb, reached the normal term of her pregnancy. Having been sent for on the 19th of September, 1857, at seven o'clock P.M., I found the vaginal portion of the cervix uteri opened for about an inch and a half, and presenting forward and upward, and rigid, and about an inch long. I prescribed unguent, belladonnæ, and endeavored at every pain to draw down the uterus, in order to bring it into the right axis. Being greatly fatigued I left the patient, leaving word to send for me as soon as any difficulty should arise.

Next morning I was called for, and heard that she had been delivered, at two A.M., of a dead boy, and that at the sexual parts a particular tumor had formed. On examination I found the whole vaginal portion of the cervix uteri torn off, and only suspended by a pedicle protruding from the vagina. Upon consultation it was decided to lay a ligature round that part of it which was undetached, and then to cut it off. This was done without causing the slightest bad symptom.

The patient did not even get the milk-fever, and felt so well, that after a few days, she left the bed, in spite of my remonstrances; in consequence of this, a hemorrhage took place several times, which, however, soon stopped. The ligature came away after four weeks.

At the beginning of April, 1858, Mrs. P. was suffering from anæmia, and I was obliged to remove a bleeding wart from her breast by ligature. About this time she became pregnant again, and was tolerably well until about the end of December, when she was again attacked with spasmodic pains of the womb. On examination I found the inner os uteri closed.

On the 3d of January, 1859, early in the morning, she had natural labor pains, and when I examined her, at one P.M., I found the inner os uteri —around the rim of which I could carry my finger—opened. The waters burst at two A.M. She was soon after delivered of a full-grown female child. The after-pains, which were very severe, were increased by her carelessness, in consequence of which she was for eight days suffering most terribly in head, limbs, and body, so severely that she had even to send for me at night time. Yet she recovered very fast, and she and her child were, when I saw her last, (May 30th, 1859,) in a perfect state of health.

---

WEDNESDAY EVENING, June 22d, 1859.

Vice-President DR. STILLÉ, in the Chair.

DR. HALL exhibited a femur, with partial fracture through the great trochanter and without the capsule, taken from the body of an adult.

DR. PACKARD presented a preparation of a larynx and trachea, completely invested with a smooth and delicately formed false membrane.

*Scirrhus of the Rectum.*—DR. LIVEZEY read the following:—

L. J., aged fifty-three, had always enjoyed good health up to his last illness, except that he complained of some irritability of the stomach, which he attributed to dyspepsia. In December last, he was attacked with pain in the lower part of the pelvis, along the course of the ureters, accompanied by pain upon pressure over the region of the kidney. He suffered at the same time from retention of urine. He was treated by a practitioner in the country by means of diuretics, and other articles, for what was supposed to be spasmodic stricture of the neck of the bladder, with an irritable or inflamed condition of that organ. The catheter was frequently used to draw off the urine. The bowels were costive; injections being required to procure a stool. In this way he remained for several months, sometimes improving, so that he could pass his urine without difficulty; at other times suffering from a relapse, when the same symptoms were presented in an aggravated form.

I saw the patient in April; he then complained very much of the irritable condition of his stomach; and about this time he had a slight hemorrhage from the urethra. Early in May he came to the city and seemed much better, although some slight œdema of the face and feet had occurred. The urine presented a straw-colored appearance, was found to be highly albuminous, with a sp. gr. of 1009; but presented nothing abnormal under the microscope. At this time a physician was called in consultation, and pronounced the case, upon careful examination by the rectum, one of cancer of the prostate gland. A few days later the patient had a return of profuse hemorrhage from the urethra. The irritable condition of stomach was increased and persistent, almost every thing being rejected, at one time even stercoraceous matter was vomited. The patient gradually became weaker until he was unable to leave his bed; the hemorrhage from the urethra recurred, and he complained of pain in and around the pelvis and lower extremities. To use his own expression, he felt as though his joints were being torn asunder. He died on the 2d instant.

Autopsy, forty-eight hours after death.—Rigor well marked.—Discoloration over the lower part of abdomen; body much emaciated; no abdominal effusion; intestines congested externally; no softening or mammillation of the mucous membrane; and no thickening or rigidity of pyloric extremity of stomach; the mesenteric glands were free from disease; the bladder was empty and contracted, its muscular coat was hypertrophied, its mucous membrane slate colored, and exhibiting signs of chronic inflammation; the prostate was enlarged about one-fourth its natural size; its internal structure was of a pale, yellowish bacony appearance. Vesiculæ seminales were healthy; the meatus was dilated. The kidneys were enlarged and quite flabby, the left one congested, but not pale or waxy looking. The pelves of the kidneys were very much distended. The rectum, about two inches above the anus, was involved in a body of cartilaginous thickening, which cut with an evident jarring to the knife. The diseased portion encircled about two-thirds of the circumference of the intestine, and extended upward about four inches; it was firmly connected with the posterior wall of the bladder, but did not appear to penetrate deeply into its structure. Under the microscope the prostate presented amorphous masses of colloid. This was tested for starch, but none was found; the cells were paler than natural. The nodular mass presented bands of fibrous tissue; but owing to the length of time that elapsed before the examination was made, it was impossible to speak definitely as to the nature of the cells. The pain in the lumbar region and the uniform diffusion of the blood through the urine, made it probable that the hemorrhage came for the most part from the kidney, at the same time that the occasional occurrence of clots in the urine, and the pain on pressure over the bladder, together with the post-mortem appearance of changes of structure in that organ, would seem to show that some at least of the blood was derived from the bladder.

*Strangulation of the Intestine.*—DR. F. G. SMITH presented, through DR. STILLÉ, the following account of a case of strangulation, with a drawing illustrative of the lesion:—

On the 19th of April, 18—, I was called to see Mr. A. W. D., who was said to be affected with a sudden attack of bilious colic, and found him suffering from constipation, nausea, restlessness, and great pain in the bowels, of a spasmodic character, but without abdominal tenderness on pressure. The usual anodynes and aperients were at once employed, but without any decided alleviation of the symptoms.

On the 20th, the nausea, griping, and restlessness had increased in violence, and distressing hiccough, with tenderness on pressure in the right iliac region, had supervened. The symptoms of local inflammatory action led to a resort to venesection, with no apparent effect, however, but that of a temporary abatement of the spasmodic pain. A few hours afterwards the pulse had become very feeble, and rated 120 beats to the minute; the singultus continued, the tenderness on pressure was increasing, and the bowels were still unmoved. Under these circumstances, a consultation with Prof. Jackson was requested, and further depletion, this time by leeches over the seat of pain, was determined on, together with the rapid administration of mercurials, with a view to their constitutional effect.

In the evening, but little improvement was observed, except in a slight lessening of the local tenderness. Early the next morning, I found him sitting on the close stool, to which he had resorted, in a vain attempt at an evacuation of his bowels. He was then pulseless, and notwithstanding immediate and most active stimulation, both externally and internally, his death followed within half an hour after he had risen from his bed. It may be stated that on the day of his attack, he had eaten rather heartily of stewed cherries; this had created some uneasiness in his bowels, on account of which he had gone to stool, and while straining was suddenly seized with a sharp pain. Domestic remedies were used for the removal of this pain, throughout the night, without effect.

Autopsy twenty-six hours after death. Present, Drs. Jackson and J. Neill.

In the cavity of the abdomen the small intestines were found to be distended with flatus, and injected, but not inflamed. Having been traced in the direction of the ilio-cæcal valve, about twelve inches of the intestine were ascertained to be completely strangulated.

The strangulation was produced in this manner: Near the termination of the ileum there was an attempt to form a supplementary cæcum, which resulted in a blind pouch communicating at a right angle with the ileum; from this abnormal pouch to the true cæcum there extended a fold of mesentery, under which a portion of the ileum had insinuated itself. During the efforts at stool, this portion of ileum probably ruptured the extended membrane, and was carried twice through the opening by the peristaltic action of the bowels. As the gut had a disposition to straighten itself, the band of membrane was twice carried around the intestine, thus most effectually strangulating it. The mode of strangulation may be demonstrated by tearing a slit, about three and a half inches long, near the edge of a piece of muslin, and then through the opening passing twice a piece of tape, doubled upon itself so as to represent a knuckle of intestine. It will be found, upon pulling on the two extremities of the tape so as to render it tight, that the edge of the muslin will embrace the tape instead of occupying its first position, thus clearly showing the manner in which the intestine was strangulated in this unfortunate gentleman's case. The strangulated portion was highly injected, but not inflamed.

The case is presented to the Society as an item of experience in a class of cases always interesting, notwithstanding their almost invariable fatality.

The Society then adjourned until September.

---

WEDNESDAY EVENING, September 14th, 1859.

The President, DR. LA ROCHE, in the Chair.

*Expectoration of Bronchial Casts.*—DR. GROSS exhibited a complete cast of a bronchial tube, with its ramifications, expectorated by a patient of Dr. James E. Reeves, of Virginia, and read Dr. Reeves's history of the case:—

Mr. J. P. T., aged thirty-five, height five feet ten inches, average weight one hundred and fifty pounds—in early life and for nine years a teamster, subsequently a dry-goods dealer—suffered a severe attack of enteric fever in the year 1853, and for twelve years, ending in 1856, was an immoderate whisky drinker. From this time he began a more temperate life, from compulsion; for during the last two years of his debauchery his health had markedly failed. He had not, as far as he could recollect, suffered from acute bronchitis.

February 23d, 1858, he requested my opinion respecting certain symptoms under which he labored, which had harassed him, with little variation, for more than a twelvemonth. These were, wandering pains in the chest and right hypochondrium; shortness of breath; frequent disposition to clear the throat, with slight huskiness of the voice; palpitations of the heart; heat and dryness of the skin; occasional flushing of the cheeks; costiveness of the bowels, and impairment of the strength and appetite.

Auscultation revealed no morbid sounds, except at the lower part of the lungs.

I advised thorough pustulation of the chest, and alteratives, under which treatment improvement seemed to follow. But on the 15th of March the patient went on military parade, the fatigue of which caused a return of his uneasy sensations, and from this date cough became more urgent. On the following day, while on the street and suffering a fit of cough, he brought up, without premonition, about two ounces of bright blood. The next day, about the same hour and at the same place, hæmoptysis recurred; eight ounces were lost. From this time up to 10th of April—when the last and most alarming hemorrhage occurred, and with it the expulsion of the casts—he continued, at intervals of from three to six days, to bring up blood in gushes; the smallest quantity brought up at any one time was two ounces; the largest, thirty; the aggregate amount lost being not less than one gallon and a half. After the second hemorrhage the blood was less florid, and frequently mixed with black coagula. As above remarked, with the expulsion of the casts the hemorrhage ceased, save an occasional bloody sputa; and from that time the patient began to improve. At present he is in the enjoyment of good health, having very much increased in flesh.

For the arrest of the hemorrhage, lead, alum, dilute sulphuric acid, gallic acid, and other astringents were used, but were of little benefit. After-treatment: Chest kept sore with tartar emetic ointment; frictions to the entire surface, night and morning, with a salt towel; small doses of morphia; cod-liver oil; syr. of iodide of iron; and milk-punch.

DR. GROSS referred to a similar case described by John Hunter.

The specimen was referred, for microscopic examination, to a committee.

